# Cognitive and Neurologic Rehabilitation Strategies for Central Nervous System HIV Infection

**DOI:** 10.1007/s11904-020-00515-0

**Published:** 2020-08-26

**Authors:** Terrence Chan, Monica Marta, Camilla Hawkins, Simon Rackstraw

**Affiliations:** 1 Mildmay Hospital, 19 Tabernacle Gardens, London, E2 7DZ UK; 2 Grahame Hayton Unit, I&I and Neurology Department, Barts Health NHS Trust, London, UK; 3 Neurosciences, Blizard Institute, Queen Mary University of London, London, UK

**Keywords:** Cognitive rehabilitation, Neuro-rehabilitation, HIV-associated neurocognitive disorders, HIV

## Abstract

**Purpose of Review:**

Cognitive impairment leading to disability is increasingly seen in people living with human immunodeficiency virus (PLWH). Rehabilitation can alleviate the effects of cognitive impairment upon function. The aim of this paper is to discuss the strategies that have been used in cognitive and neurologic rehabilitation in PLWH.

**Recent Findings:**

Studies examining pharmacological and non-pharmacological strategies were analysed. Medical management of HIV and co-morbidities should be optimised. Non-pharmacological strategies, including nerve stimulation techniques, exercise-based interventions, and paper and computer-based cognitive rehabilitation, have some evidence supporting their use in PLWH either as stand-alone interventions or as part of a multidisciplinary approach.

**Summary:**

Both pharmacological and non-pharmacological rehabilitation strategies have been used with PLWH. More intervention trials are needed to assess cognitive and neurological rehabilitation strategies and further evaluate their potential benefit in PLWH.

## Introduction

HIV-associated neurocognitive disorder (HAND) was characterised by severe cognitive dysfunction leading to dementia and death in the pre-antiretroviral era [1]. The introduction of combination antiretroviral therapy (cART) decreased the incidence of the most severe HAND cases [2–4]. It also transformed the management of HIV infection from the symptomatic control of a life-limiting disease to the care of a chronic condition that is associated with a close to normal lifespan [5].

Whilst the most severe forms of HAND are now rare, milder cognitive impairments persist amongst PLWH [3, 6, 7]. Recent study cohorts suggest that cART does not alter the prevalence of cognitive impairment in PLWH [6, 8, 9]. Furthermore, new HIV-related neurological complications have become apparent with the widespread use of cART. Encephalopathy due to HIV discordance between plasma and cerebrospinal fluid (CSF) [10–12] and CD8 encephalitis [13] is rare but recognisable central nervous system (CNS) presentations seen in PLWH on cART. It has also emerged that some antiretrovirals, such as efavirenz, have direct CNS toxicity [14, 15]. HIV infection is also associated with a higher risk of cerebro- and cardiovascular disease, either by lifestyle, direct viral or antiretroviral effects [16] (e.g. hyperlipidaemia caused by protease inhibitor–based therapy). Cerebrovascular disease is associated with an increased prevalence of neurological disability and cognitive difficulties. As the life expectancy for PLWH increases due to improved medical management, it has become apparent that some PLWH with neurological and cognitive impairments may survive for many years following acute or ongoing neurological insults.

Rehabilitation strategies are needed to improve quality of life, day to day functional performance and to mitigate further cognitive impairment. Neurological rehabilitation can be defined as a process that aims to optimise health, social functioning and sense of well-being through restorative and compensatory strategies [17]. This definition highlights that rehabilitation should be a personalised intervention. It is not a process restricted to people who may recover, partially or completely, but applies to all.

The London Taxi driver study [18•] showed that neuronal volumes can be influenced by tasks, demonstrating the concept of neuroplasticity following interventions. Neurological rehabilitation strategies, particularly those involving multidisciplinary teams (MDT), have been traditionally used in cohorts of people with more static brain injuries, such as traumatic brain injury, stroke or post-hypoxic brain injury [19] but also increasingly in chronic diseases such as multiple sclerosis [20]. These teams include physicians and psychiatrists, rehabilitation nurses, physiotherapists, speech and language therapists, occupational therapists and psychologists.

In this review, we discuss the strategies that have been used in cognitive and neurologic rehabilitation in PLWH.

## Methods

We reviewed outcomes data in our neuro-rehabilitation unit for PLWH.

We used PubMed, Embase, CINAHL, and the Psychology and Behavioural Sciences databases to search for the terms ‘HIV’, ‘cognitive’, and ‘neurological’ and ‘rehabilitation’. We reviewed 892 abstracts and selected studies in English that had a clear rehabilitation intervention and described the outcomes in PLWH.

## Results

Whilst many publications looked at cognitive and neurological disability and rehabilitation techniques, the majority did not describe the analysis of an intervention: most of these studies were not randomised, had only small numbers of PLWH and were pilot studies.

We have highlighted studies that demonstrate particular rehabilitation strategies that best illustrate the changes experienced by PLWH following the intervention.

### Multidisciplinary Therapy Team Strategies—the Example of Mildmay Hospital

The Mildmay hospital is an inpatient and day therapy unit in London which is dedicated to the rehabilitation of people with neurological disability caused by or associated with HIV infection. With the use of cART, it evolved from a palliative care unit for people dying with HIV infection to a rehabilitation unit. In the pre-cART era, the brain impairment unit gained experience of delivering neurological and cognitive assessment and care to PLWH admitted for long-term care. Those admitted were very immunocompromised and had neuro-behavioural problems, with severe cognitive impairment secondary to HIV-related neurological disorders (e.g. HIV encephalopathy), opportunistic infections (e.g. cerebral toxoplasmosis or progressive multifocal leukoencephalopathy) or brain tumours. A combination of rehabilitation techniques and cART led to patients with HIV-related brain pathology being discharged with better function rather than dying within the unit [21•].

Currently, PLWH admitted to Mildmay Hospital have been diagnosed with HAND or with cognitive and physical impairments due to neurological opportunistic infections or other HIV-related brain pathology. They tend to have been diagnosed late with HIV infection or have had poor adherence to cART. PLWH are assessed by an MDT that includes an HIV physician, psychiatrist, physiotherapist, occupational therapist, dietitian, social worker, speech and language therapist and psychologist, who design a personalised programme of rehabilitation to meet individual needs and goals.

**Fig. 1:**
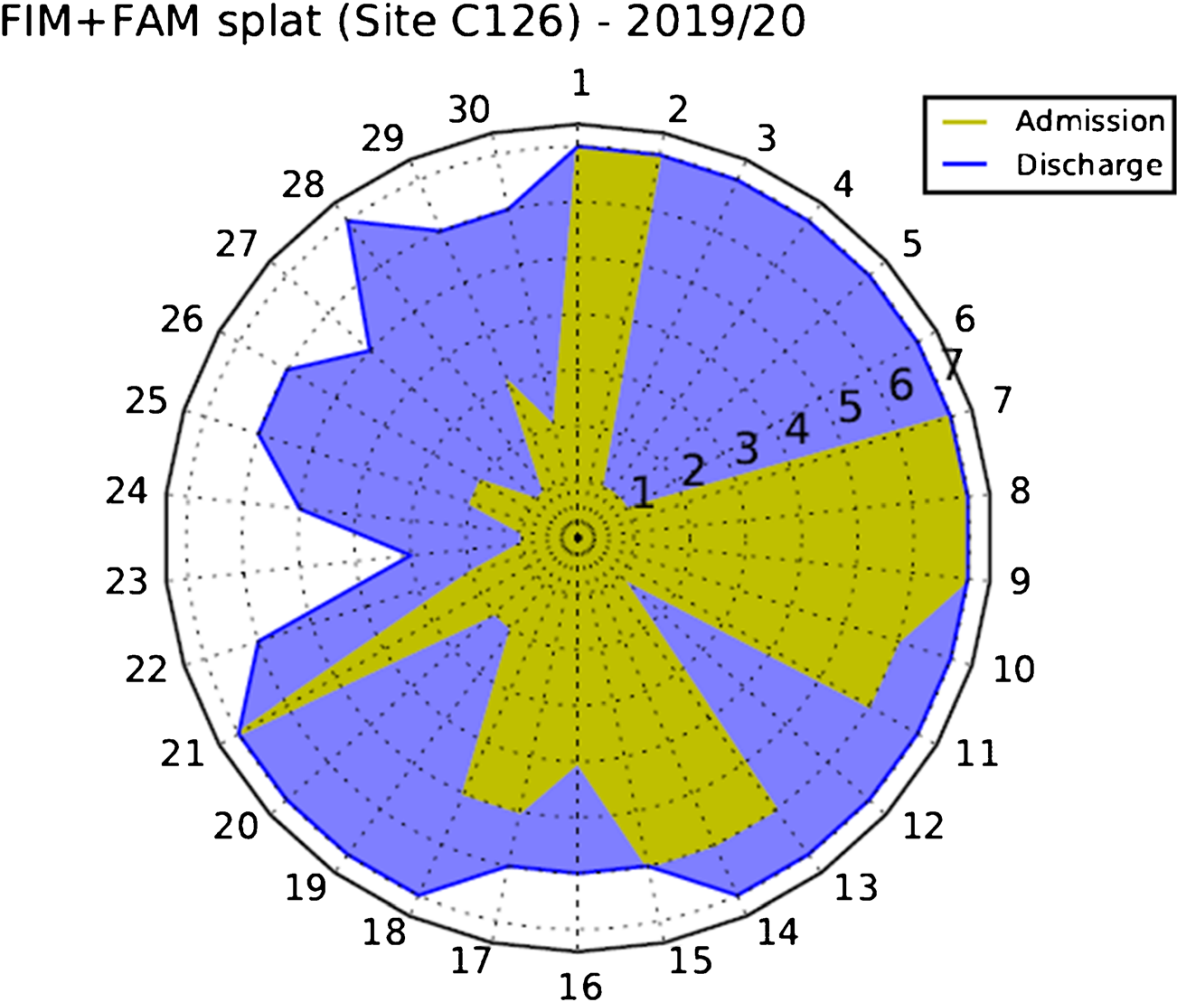
Splat-plot of FIM + FAM domain admission and discharge scores in 2019. FIM, functional independence measure; FAM, functional assessment measure

We measure change during rehabilitation with the Functional Independence Measurement (FIM) and Functional Assessment Measure (FAM) scales adapted for use in the United Kingdom (UK) [22, 23]. These are the principal outcome measure for the UK national database for specialist rehabilitation in patients with complex disabilities [24] (the UK Rehabilitation Outcomes Collaborative (UKROC)). The FAM was developed in the early 1990s for people with traumatic brain injury and includes 12 items that focus on cognitive and psychosocial function [23]. It is used in combination with the 18-item FIM at admission and discharge. The outcomes can be viewed in the form of a ‘splat-plot’ that change from admission to discharge by the differential area covered, with higher scores indicating better function. An example of the outcomes can be seen in Fig. 1 and Table 1.

**Table 1:** Functional gains achieved with multidisciplinary inpatient rehabilitation

	Site C126			
	2017/18	2018/19	2019/20	Total
Complexity (serial RCS-E scores)				
RCS-E v12 score (mean)	11.2	11.4	11.1	11.3
% High/very high assessments	68.0%	64.6%	60.9%	65.5%
Functional gain (FIM + FAM scores)				
% reported	61%	64%	25%	60%
Motor score on admission (mean)	90.7	87.9	67.3	88.7
Motor score on discharge (mean)	99.2	96.7	79.0	97.5
Motor gain during episode (mean)	8.5	8.9	11.7	8.8
Cognitive score on admission (mean)	70.9	72.8	46.3	71.0
Cognitive score on discharge (mean)	77.3	80.7	65.3	78.5
Cognitive gain during episode (mean)	6.5	7.9	19.0	7.5
FIM + FAM efficiency (median)	0.2	0.1	0.0	0.1
FIM + FAM efficiency (pop. mean)	0.2	0.2	0.4	0.2
Reduction in cost of ongoing care				
% reported (optional)	34%	51%	75%	44%
Mean NPCNA cost/week on admission	£1216	£706	£606	£894
Mean NPCNA cost/week on discharge	£548	£392	£73	£413
Mean saving in cost/week	£668	£314	£532	£481
NPCNA cost efficiency index (median)	18.4	1.5	4.0	3.8
NPCNA cost efficiency index (pop.median)	7.4	4.7	7.2	6.1

An advantage of this multidisciplinary approach to rehabilitation and its evaluation is that it focuses on measuring functional, more ecological outcomes, rather than neurocognitive test scores, and may have more validity to people’s daily lives.

### ‘Medical Maximisation’ Strategies with cART

Although it is now widely accepted that all PLWH regardless of stage of disease should be commenced and maintained on cART [25], this has not always been the case. Due to the toxicities of older regimes, cART was mostly used to treat patients with symptomatic immunosuppression.

Many PLWH benefited cognitively when started on highly active cART, with a global improvement in cognitive domains affected by the disease [8, 26, 27]. On the other hand, there were occasional examples of cohorts that improved cognitively by stopping cART, suggesting a toxicity effect of some medications [28]. Based on this evidence, treatment guidelines suggested that all patients with HIV-related cognitive impairment should be placed on cART, even at a time when they were unable to make such a recommendation for all patients [29].

Pharmacodynamics and pharmacokinetics of individual ART medications within the CNS differ when compared with the systemic circulation. This underpinned the hypothesis that some drugs lack efficacy in the CNS due to a decreased drug penetration in the CNS compartment and the concept of an ARV regimen CNS Penetration-Effectiveness (CPE) score (the higher the score, the better the CNS penetration) [30]. A number of studies have used this ranking system to examine whether regimes with higher CPE scores have an impact upon PLWH’s cognition but the results have been inconsistent, with both positive and negative results reported [31–34].

Another strategy was the addition of maraviroc to the regimes of patients with cognitive impairment. Maraviroc has an effect on neuro-inflammation and monocyte activity, and was used as an adjunct in virologically suppressed patients, with some success in small pilot studies [35, 36]. Other cART optimisation strategies include a switch from regimes that have been associated with poor neurocognitive performance, particularly efavirenz. Neuropsychological symptoms, and in some cases objective neuropsychological dysfunction, have been noted as side effects of using efavirenz in cART regimes [15], and switching from efavirenz-containing regimes has been associated with improved well-being but not always cognitive benefit in formal testing [37].

Recently, a differentially resistant virus in CSF and plasma (HIV compartmentalisation) has been found in PLWH with neurologically symptomatic disease. Although symptomatic HIV CSF escape is rare [10, 11], modification of cART according to the genotypes of both plasma and CSF compartments may improve cognitive symptoms amongst these patients.

### ‘Medical Maximisation’ Strategies—Management of Co-Morbidities

There continues to be some debate about the aetiology of cognitive difficulties in HIV infection and the extent to which other medical problems that are affecting PLWH also compound the effects of HAND [3].

Co-infections have been associated with neurocognitive impairments, and therefore require effective management. Hepatitis C infection is associated with neurocognitive impairments in both mono-infected patients and in those co-infected with HIV infection [38, 39]. There have been some studies demonstrating some neurocognitive benefit in virological clearance of hepatitis C with both interferon and direct acting agents [40, 41].

Poorly controlled cardiovascular risk factors such as diabetes or hypertension are associated with poorer cognitive outcomes in PLWH [36–39]. Studies in improved management of those diseases have shown mixed results in improving cognition [42].

Many cohort studies show high levels of psychiatric disorders in PLWH. Depression and anxiety can mask or resemble symptoms of cognitive impairment. In addition, severe mental health difficulties may affect performance in neurocognitive tests and the ability of PLWH to perform day to day tasks. There is some evidence that neurocognitive functions can be improved by active treatment of depression with some antidepressants [43].

There has also been interest in the treatment of HAND by the addition of other adjunctive medications to try to ameliorate the cytokine changes associated with HAND [44–47]. The effects in pilot studies have been variable, but usually disappointing, showing very little real cognitive change.

### Nerve Stimulation Techniques

A number of different nerve stimulation techniques have been proposed as adjunctive methods as part of neuro-rehabilitation. Techniques such as binaural beat therapy have been used experimentally to treat anxiety and improve cognitive processing. Similarly, vagal nerve stimulation has been examined as a treatment for both refractory depression and epilepsy: some of the patients treated in these studies had some improvements in attention and memory recall. Neither of these techniques has yet been used in PLWH.

However, transcranial direct current stimulation has been trialled in PLWH. Electrodes are placed cutaneously over parts of the scalp corresponding to neural networks, and are thought to influence change in neuronal activity or neurotransmitter release. This was tested in a driving simulation study, combined with speed of processing cognitive remediation therapy in a randomised 1:1 fashion between two groups of 15 unimpaired PLWH. Results suggested more cautious driving behaviour following active intervention. People in the training group showed fewer lane deviations and a slower average driving speed, in contrast to the control group who showed an increase in driving deviations and average driving speed [48].

A combination of techniques was tested further in a group of 33 older (> 50 years) PLWH. The participants underwent neuropsychological testing and training with combination speed of processing cognitive remediation therapy, and were then randomised to receive either real transcranial direct stimulation (*n* = 17) or not (*n* = 16). The neuropsychological testing after the intervention showed small-to-medium effects in the executive and speed of processing measure. Medium-to-large effects were observed for an executive/attention and oral reading measure. There were small-to-medium and medium-to-large effects for two speed of processing measures in the opposite direction (i.e. the control group showed greater improvements). However, the only statistically significant effect was seen on the oral reading measure [49]. It is a technique that needs further studies to examine it further as part of a neuro-rehabilitation strategy.

### Exercise-Based Interventions

Exercise- or physical activity–based interventions were used to both improve cognitive abilities, or to prevent cognitive decline in PLWH. There is already a body of data attesting to the benefits of exercise on cognition in non-HIV populations [50, 51].

A small study randomised 11 PLWH who were not cognitively impaired at baseline into either an exercise group (*n* = 5) who completed a 16-week aerobic exercise programme training 3 times per week or a control non-exercise group (*n* = 6). The results showed higher moderate physical activity correlated with higher MOCA scores, but exercise training did not induce significant improvements in cognitive function [52]. It is important to note that adherence to the intervention was only 60%.

Yoga as a strategy for cognitive improvement was tested in 18 adolescent PLWH at a rehabilitation centre in India. It was a single-group study with a pre- and post-intervention assessment of general health, immune parameters, quality of life and cognitive functioning. All the participants were all given 1 h of yoga daily for 6 months. The results demonstrated an improvement in general health of the participants. However, the cognitive function analysis had mixed results with improved psychomotor performance but worsened executive functioning [53]. This is a strategy which has seen some promising results in the general older population and deserves to be studied further.

### Paper-Based Cognitive Rehabilitation

A study from San Diego used Goal Management Training (GMT) and metacognitive training as a strategy for improving executive function difficulties, seen in people with HAND and in people with substance use. GMT showed efficacy in improving executive function difficulties in daily life. Metacognition or ‘thinking about thinking’ is based on an active awareness of cognitive functions and understanding of cognitive difficulties, which is also often suboptimal in those with HAND and those who use substances.

Ninety PLWH with a previous or current history of substance use and current executive function difficulties were randomised 1:1:1 to either receive a single session of Goal Management Training or a single session of both Goal Management Training plus Metacognitive Training or to an active control group, with 30 participants in each group [54]. The results suggest that a brief, single session of Goal Management Training (GMT) protocol has some effect on multitasking abilities.

An Italian study is an example of the combination of paper-based and computerised cognitive–based exercises as a cognitive rehabilitation strategy. Thirty-two PLWH (16 with and 16 without HAND) were randomised 1:1 to the intervention (eight different exercises, lasting about 50 min in duration, which were repeated 36 times over a 4-month period) or as a control. Neurocognitive assessment was performed before, and after the study in all participants, and six months after the completion of the intervention for those in the interventional arm. The intervention was directed at improving abilities within four cognitive domains: attention, visual-verbal memory and learning, executive functioning and working memory, and metacognitive awareness. The intervention group demonstrated improvements in five of eight cognitive domains measured, including learning and memory, abstraction/executive function, verbal fluency, attention, and functional tasks, whereas those in the control group showed a deterioration in those domains. When re-measured after 6 months, the intervention group had maintained improvements in abstraction/executive functioning, attention/working memory and functional tasks [55••]. This demonstrates that patients can participate in a cognitive rehabilitation protocol, make cognitive gains and maintain them, including in functional tasks 6 months after completion of the programme.

### Computer-Based Strategies

Computerised neuropsychological assessments have become increasingly common in recent years. There has been particular interest in brief screens and their use in intervention studies. This is because of the speed of execution and decreased cost compared with traditional full neuropsychological assessments. They have been validated in several disease areas including in PLWH with various degrees of cognitive impairment. There has also been some interest in using computerised cognitive rehabilitation programmes, as these potentially have the advantage of being able to be performed in the community without supervision. A couple of studies have shown some potential advantages and disadvantages of the approach.

In a partially randomised study of 30 PLWH and 30 HIV-negative adults, those assigned to the intervention group were asked to use SmartBrain (SmartBrain Technologies, 2013) from their home computer for 24 weeks, initially for 10 min and increasing weekly to a maximum of 30 min [56]. SmartBrain contains 14 game-like modules in the domains of memory, attention, knowledge and executive functions. Participants were tested with an extensive battery of neurocognitive assessments before and after the intervention. Primary results from this study revealed no significant effects of SmartBrain on global cognitive outcomes, irrespective of HIV serostatus. However, adherence to the intervention was poor with only 54% of participants able to use the programme more than once. A post hoc analysis showed that the most benefit was gained by those who undertook the intervention most frequently.

In Uganda, 159 6 to 12-year-old HIV positive children were randomised 1:1:1 to a group either receiving a computerised rehabilitation programme using computer games that was progressively more difficult, to a limited programme that generated games of random degrees of difficulty, or no computerised intervention, over a 2-month period [57••]. The computerised rehabilitation package was called Captain’s Log (BrainTrain Corporation) programmed with games to improve working memory, attention and visual–spatial analysis. Planning and knowledge domains had significantly greater gains at follow-up in the full intervention group when compared with controls. Both intervention arms had significant improvements on learning tests compared with controls. This demonstrates that this could be a strategy for use in resource-poor country situations.

## Conclusion

Cognitive impairment is still prevalent in PLWH in the cART era, and can lead to disability affecting activities of daily living. Both pharmacological and non-pharmacological rehabilitation strategies are currently being increasingly used to minimise the effect of such impairments. Early studies suggest promise in remediating some of the cognitive impairment caused by HIV. More intervention trials are needed to assess cognitive and neurological rehabilitation strategies and further evaluate their benefit in PLWH.
